# Prevalence of tuberculosis, hepatitis C virus, and HIV in homeless people: a systematic review and meta-analysis

**DOI:** 10.1016/S1473-3099(12)70177-9

**Published:** 2012-11

**Authors:** Ulla Beijer, Achim Wolf, Seena Fazel

**Affiliations:** a Department of Women's and Children's Health, Karolinska Institutet, Astrid Lindgren Children's Hospital, Stockholm, Sweden; b Division of Social Medicine, Department of Public Health Sciences, Karolinska Institutet, Stockholm, Sweden; c Department of Psychiatry, University of Oxford, Warneford Hospital, Oxford, UK; d Oxford Health NHS Foundation Trust, Oxford, UK

## Abstract

**Background:**

100 million people worldwide are homeless; rates of mortality and morbidity are high in this population. The contribution of infectious diseases to these adverse outcomes is uncertain. Accurate estimates of prevalence data are important for public policy and planning and development of clinical services tailored to homeless people. We aimed to establish the prevalence of tuberculosis, hepatitis C virus, and HIV in homeless people.

**Methods:**

We searched PubMed, Embase, and Cumulative Index to Nursing and Allied Health Literature for studies of the prevalence of tuberculosis, hepatitis C virus, and HIV in homeless populations. We also searched bibliographic indices, scanned reference lists, and corresponded with authors. We explored potential sources of heterogeneity in the estimates by metaregression analysis and calculated prevalence ratios to compare prevalence estimates for homeless people with those for the general population.

**Findings:**

We identified 43 eligible surveys with a total population of 63 812 (59 736 homeless individuals when duplication due to overlapping samples was accounted for). Prevalences ranged from 0·2% to 7·7% for tuberculosis, 3·9% to 36·2% for hepatitis C virus infection, and 0·3% to 21·1% for HIV infection. We noted substantial heterogeneity in prevalence estimates for tuberculosis, hepatitis C virus infection, and HIV infection (all Cochran's χ^2^ significant at p<0·0001; *I*^2^=83%, 95% CI 76–89; 95%, 94–96; and 94%, 93–95; respectively). Prevalence ratios ranged from 34 to 452 for tuberculosis, 4 to 70 for hepatitis C virus infection, and 1 to 77 for HIV infection. Tuberculosis prevalence was higher in studies in which diagnosis was by chest radiography than in those which used other diagnostic methods and in countries with a higher general population prevalence than in those with a lower general prevalence. Prevalence of HIV infection was lower in newer studies than in older ones and was higher in the USA than in the rest of the world.

**Interpretation:**

Heterogeneity in prevalence estimates for tuberculosis, hepatitis C virus, and HIV suggests the need for local surveys to inform development of health services for homeless people. The role of targeted and population-based measures in the reduction of risks of infectious diseases, premature mortality, and other adverse outcomes needs further examination. Guidelines for screening and treatment of infectious diseases in homeless people might need to be reviewed.

**Funding:**

The Wellcome Trust.

## Introduction

An estimated 100 million people worldwide are homeless.^1^ In high-income countries, country-specific data suggest that more than 650 000 individuals in the USA^2^ and around 380 000 in the UK^3^ are homeless at any one time. Although most live in sheltered accommodation—eg, emergency hostels, bed and breakfasts, squats, or other temporary accommodation—a 2011 US report^2^ has estimated that 39% of homeless people are unsheltered, and thus roughly 250 000 individuals live on the streets, more than 120 000 of whom are in the New York City and Los Angeles metropolitan areas.^2^ Although methodological difficulties exist in counting homeless people and definitions of homelessness vary, these estimates help to quantify the number of homeless people.^4^

Health problems in homeless populations have been previously reported.^5^, ^6^ Mortality rates are four times higher than in the general population.^7^ Morbidity is substantially increased in homeless populations, who have higher prevalences of mental disorders^8^ and infectious diseases than do general populations,^6^ which, being modifiable, could be targeted by health interventions to reduce the frequency of adverse outcomes. Infections in homeless people can lead to community infections and are associated with malnutrition,^9^ long periods of homelessness,^10^ and high use of medical services.^11^ Because absolute numbers of homeless people are high in some countries, improvements in care could have pronounced effects on public health.

A wide range of estimates for the prevalence of infectious diseases in homeless people have been reported, particularly for tuberculosis, hepatitis C virus, and HIV.^12^ A synthesis of these prevalence data would be important for public policy and planning and development of clinical services addressing the needs of homeless people. It could also inform future projects by identifying methodological problems and research priorities.

We did a systematic review and metaregression analysis to establish the prevalence of tuberculosis, hepatitis C virus, and HIV in homeless people. We explored by metaregression the reasons for variations between the primary studies and examined whether prevalence varied by year of publication, sex, study region, diagnostic method, and study size.

## Methods

### Search strategy and selection criteria

We searched PubMed, Embase, and Cumulative Index to Nursing and Allied Health Literature with the term “homeless* and (tuberculosis or HIV or hepatitis C or HCV)” for studies of the prevalences of tuberculosis and hepatitis C virus and HIV infections in homeless people published between Jan 1, 1980, and Jan 31, 2012. We focused on these diseases after preliminary searches showed many reports estimating their prevalences and reviews emphasising their importance.^6^, ^12^ We also searched relevant reference lists and relevant journals by hand and corresponded with authors. We translated non-English-language articles. Our analyses accorded with the preferred reporting items for systematic reviews and meta-analyses (PRISMA) guidelines (when appropriate) for a systematic review of prevalences.^13^

Studies had to meet two criteria for inclusion. They had to investigate adults with no fixed abode (owned or rented), who rely on temporary accommodation, live in institutions or shelters, or live rough (in a context where most peers have homes and homelessness is not because of war, conflict, or natural disasters), and had to present data that allowed for establishment of prevalences of tuberculosis, hepatitis C virus, and HIV. Investigations were included irrespective of diagnostic methods, but mostly included chest radiography for tuberculosis and blood tests for hepatitis C virus, and HIV. Diagnoses based on questionnaires (ie, self-report of disease status) were also included.

We excluded reports if the number of homeless individuals was not reported separately from the number of non-homeless people and could not be obtained from the authors; prevalences of infectious diseases were grouped and not available separately for tuberculosis, hepatitis C virus, and HIV; the methods were unclear; or the study population was homeless drug users only (drug use is a major risk factor for infection with HIV and hepatitis C virus,^12^ and prevalence would therefore not be representative of homeless populations). Bucher and colleagues' study^14^ investigated both homeless people and individuals who live in single-room occupancies. However, we included only the homeless subgroup in our analysis—we did not deem those in single-room occupancy homeless.

Two reviewers (Anna Francis [Oxford University Medical School, Oxford, UK] and UB for most studies; AW and UB for the remainder) independently extracted information about geographical location, year of publication, definition of homelessness, duration of homelessness, risk factors for homelessness, method of sample selection, sample size, mean age, diagnostic method, diagnostic criteria, and numbers diagnosed with tuberculosis, hepatitis C virus, or HIV from every eligible study. Disagreement was resolved by consensus between the two reviewers or through consultation with the corresponding author, when necessary. If needed, we sought further clarifications from the authors of relevant studies.

### Statistical analysis

We calculated prevalence estimates with the variance-stabilising double arcsine transformation,^15^ because the inverse variance weight in fixed-effects meta-analyses is suboptimum when dealing with binary data with low prevalences. Additionally, the transformed prevalences are weighted very slightly towards 50%, and studies with prevalences of zero can thus be included in the analysis. We used the Wilson method^16^ to calculate 95% CIs around these estimates because the asymptotic method produces intervals which can extend below zero.^17^ We estimated heterogeneity between studies with Cochran's Q (reported as χ^2^ and p values) and the *I*^2^ statistic, which describes the percentage of variation between studies that is due to heterogeneity rather than chance.^18^, ^19^ Unlike Q, *I*^2^ does not inherently depend on the number of studies included; values of 25%, 50%, and 75% show low, moderate, and high degrees of heterogeneity, respectively. Because heterogeneity was high (*I*^2^ >75%), we used random-effects models for summary statistics.^19^ These models (in which the individual study weight is the sum of the weight used in a fixed-effects model and between-study variability) produce study weights that mainly show between-study variation and thus provide close to equal weighting. In our analyses, we split study populations into male and female groups as appropriate. We defined studies as mixed when only overall estimates of the prevalences of infection were reported and we could not obtain further information from the authors to stratify results by sex. In a subgroup analysis, we calculated the prevalences of tuberculosis, hepatitis C, and HIV for US and European studies to provide estimates for high-income countries and to allow comparison with studies of prevalence done in prisoners in those geographical regions. We did three sensitivity analyses; we excluded one large study^20^ from the tuberculosis group and, for both hepatitis C virus^21^, ^22^ and HIV infections,^21^, ^22^, ^23^, ^24^, ^25^, ^26^, ^27^ we looked only at studies of prevalence that used blood samples for diagnosis.

We did an additional analysis that compared the prevalences in homeless people with estimated prevalences in the general population to calculate prevalence ratios and 95% CIs. Information about the prevalence of tuberculosis and HIV infection in the general population was obtained from the UN Millennium Development Goals Database.^28^, ^29^ For hepatitis C virus infection, we used country-specific estimates from US and European studies.^30^, ^31^ We obtained denominator (total population) data from the UN World Population Prospects Database.^32^ We chose the national data that most closely matched the year of publication of the study.

### Heterogeneity

We further investigated potential sources of heterogeneity by arranging groups of studies according to potentially relevant characteristics and by metaregression analysis, which attempts to relate differences in effect sizes to study characteristics.^33^ Factors examined both individually and in multiple-variable models were year of publication, sex (by comparing mixed-sex samples with single-sex samples), geographical region (by comparing USA-based studies with those based elsewhere), study size (by comparing investigations of more than 500 individuals with smaller studies, and additionally through use of sample size as a continuous variable), diagnostic method (by comparing studies that diagnosed tuberculosis by chest radiography with reports that used other methods, and studies that used blood analysis for diagnosis of HIV or hepatitis C virus infections with those that diagnosed by other methods), and estimates of prevalence in the general population of the study country (as a continuous variable). These factors were selected on the basis of previous work about heterogeneity in prevalence studies in homeless people.^34^ We entered only factors that we deemed significant individually (p<0·05) into a multiple regression model to avoid model instability. The regression coefficients for each study characteristic on individual analysis were provided to enable comparison across diagnoses. We did all analyses in Stata (version 12·1) with the commands metan (for random-effects meta-analysis specifying three variables: double-arcsine-transformed prevalence, Wilson CIs, and prevalence ratios) and metareg (for metaregression).

### Role of the funding source

The Wellcome Trust had no role in study design, data collection, analysis, interpretation, or writing of the report. All authors had full access to the study data and had final responsibility for the decision to submit for publication.

## Results

Our searches returned a total of 4024 records (appendix). After removal of duplicates and initial screening, we reviewed 210 papers in full. After exclusion of ineligible reports, our final sample was 43 studies (n=63 812) published between December, 1984, and Jan 12, 2012, including 17 studies (43 605) of tuberculosis^9^, ^20^, ^35^, ^36^, ^37^, ^38^, ^39^, ^40^, ^41^, ^42^, ^43^, ^44^, ^45^, ^46^, ^47^, ^48^, ^49^ (table 1), 12 (5391) of hepatitis C^21^, ^22^, ^50^, ^51^, ^52^, ^53^, ^54^, ^55^, ^56^, ^57^, ^58^, ^59^ (table 2), and 22 (14 816) of HIV^10^, ^14^, ^21^, ^22^, ^23^, ^24^, ^25^, ^26^, ^27^, ^50^, ^52^, ^53^, ^54^, ^55^, ^59^, ^60^, ^61^, ^62^, ^63^, ^64^, ^65^, ^66^ (table 3). After taking into account duplication of patients because of overlapping samples, the overall population consisted of 59 736 homeless individuals.

**Table 1:** Studies of the prevalence of tuberculosis in homeless people

	**Country**	**Sex**	**Sampling method**	**Diagnostic method**	**Mean age**	**n**
Glicksman et al,^38^ 1984	USA	Male	Homeless men in one shelter for men and two hotels in New York City, NY	Unknown	..	198
Barry et al,^9^ 1986	USA	Male	Homeless men in three large shelters in Boston, MA during four nights	Chest radiography	..	465
Capewell et al,^37^ 1986	UK	Male	Adult homeless men in eight hostels or shelters in Edinburgh, Scotland	Chest radiography	..	2150
Ramsden et al,^44^ 1988	UK	Mixed	Two centres for homeless people in London	Chest radiography	..	555
Kumar et al,^42^ 1995	UK	Male	All homeless people in a shelter in London	Chest radiography	41	557
Kimerling et al,^41^ 1999	USA	Male	Homeless men in two shelters in Birmingham, AL	Sputum	41	127
Southern et al,^47^ 1999	UK	Male	Homeless adults in hostels, night shelters, and day centres for homeless in London	Chest radiography	..	1905
Moss et al, ^43^ 2000*	USA	Mixed	Homeless adults from free food lines and shelters in San Francisco, CA	Sputum	38	2764
Zunic et al,^49^ 2000	France	Mixed	Homeless adults in shelters in Paris	Chest radiography	..	663
Solsona et al,^46^ 2001	Spain	Male	Homeless people admitted to shelters for homeless people or soup kitchens, or both	Chest radiography	..	394
Kern et al,^40^ 2005	France	Mixed	Homeless adults in shelters in Paris	Chest radiography	..	204
Romaszko et al,^45^ 2008†	Poland	Mixed	Social service workers were trained to reach out to homeless people	Chest radiography	..	305
Badiaga et al,^35^ 2009‡	France	Male	All homeless people in two shelters in Marseilles	Chest radiography	41	208
Beijer et al,^36^ 2009§	Sweden	Mixed	All homeless adults (in shelters, temporary accommodations, etc) who were documented as homeless in 1996	Unknown	34	1704
McAdam et al,^20^ 2009	USA	Male	Eight shelters and drop-in centres in New York City, NY	Unknown	43	28 835
Tabuchi et al,^48^ 2011	Japan	Male	Homeless people associated with the shelter and soup-run in Airin district, Osaka	Chest radiography	58	263
Goetsch et al,^39^ 2012	Germany	Mixed	Homeless people in shelters or facilities for homeless people in Frankfurt	Chest radiography	41	2308

* HIV infection and being older than 50 years were positively associated with prevalence; use of injection drugs, crack cocaine, or alcohol was not significantly associated with prevalence.

† 67% of participants were homeless for greater than 3 years.

‡ Participants were homeless for <6 months (43%), 7–12 months (12%), 13–24 months (9%), or >24 months (36%).

§ Participants' sex was not significantly associated with prevalence.

**Table 2:** Studies of the prevalence of hepatitis C virus infection in homeless people

	**Country**	**Sex**	**Sampling method**	**Inclusion criteria**	**Diagnostic method**	**Mean age**	**n**	**Length of homelessness**	**Risk factors**
Rosenblum et al,^55^ 2001	USA	Mixed	Homeless people in contact with mobile clinic, Manhattan, New York City, NY	Homeless, 21–58 years old	Blood	40	139	..	Use of injection drugs was positively associated with prevalence
Nyamathi et al,^54^ 2002	USA	Female	Derived from a large study of homeless women in shelters and on the streets, Los Angeles, CA	Homeless, 18–65 years old, having an intimate partner or friend willing to participate	Blood	..	884	..	Use of injection drugs was positively associated with prevalence
Sherriff et al,^56^ 2003	UK	Mixed	Homeless people from shelters, special projects, and medical centres in Oxford	Homeless adults	Oral fluid	..	98	..	Use of injection drugs and sharing of drug paraphernalia were positively associated with prevalence; age, needle sharing, alcohol, sexual activity, family history of hepatitis C virus infection, tattoos, and piercings were not significantly associated
Beijer,^50^ 2007	Sweden	Mixed	All homeless people in contact with the Social Services Unit for Homeless People in Stockholm	Homeless adults	Blood	42	2285	..	..
Brito et al,^52^ 2007	Brazil	Mixed	Homeless adults who use shelters, São Paulo, Brazil	Homeless adults aged 18 years or older without psychiatric disturbances who use shelters	Blood	40	330	<1 year (39%), >5 years (13%)	Use of injection drugs, sharing of drug paraphernalia, and previous imprisonment were positively associated with prevalence
Burström et al,^21^ 2007	Sweden	Mixed	Homeless adults in shelters and institutions and rough sleepers in Stockholm	Homeless adults	Questionnaire	48	155	>10 years (40%)	..
O'Carroll et al,^22^ 2008	Ireland	Male	All homeless people living in temporary accommodation in Dublin	Homeless adults in hostels and bed and breakfasts	Questionnaire	..	343	..	..
Schwarz et al,^58^ 2008	USA	Female	Homeless families or caregivers with children in shelters and transitional houses, Baltimore City, MD	Homeless adults or caregivers with children	Blood	..	161	..	..
Boyce et al,^51^ 2009	USA	Mixed	Homeless shelter in Honolulu, HI	Homeless people in a shelter	Blood	39	40	..	..
Vahdani et al,^59^ 2009	Iran	Male	Homeless people in institutions of the municipal authorities, shelter homeless, and rough sleepers in Tehran	Homeless, in a shelter	Blood	45	202	Mean 502 days (range 10–3700)	..
Colson et al,^53^ 2011	France	Male	Two homeless shelters in Marseilles	Homeless adults	Blood	41	220	..	..
Stein et al,^57^ 2011	USA	Mixed	Homeless people in shelters and meal programmes, Los Angeles, CA	Homeless adults	Blood	..	534	..	..

**Table 3:** Studies of the prevalence of HIV infection in homeless people

	**Country**	**Sex**	**Sampling method**	**Inclusion criteria**	**Diagnostic method**	**Mean age**	**n**	**Length of homelessness**	**Risk factors**
Zolopa et al,^10^ 1994	USA	Mixed	Random sampling from shelters and soup kitchens in San Francisco, CA	Homeless adults older than 18 years	Blood	36	1226	Median 12 months in men, 6 months in women (range 1 day–40 years)	Injection drug use, black race, homosexuality, and sex work were positively associated with prevalence; age was not significantly associated
Paris et al,^64^ 1996	USA	Mixed	Homeless people in contact with mobile outreach team in Atlanta, GA	Homeless, in contact with a mobile outreach team or clinic	Blood	..	535	..	..
Magura et al,^63^ 2000	USA	Mixed	Homeless men and women from two soup kitchens in New York City, NY	Homeless adults	Blood	..	191	..	..
Rosenblum et al,^55^ 2001	USA	Mixed	Homeless people in contact with mobile clinic, Manhattan, New York City, NY	Homeless, 21–58 years old	Blood	40	139	..	..
Nyamathi et al,^54^ 2002	USA	Female	Derived from a large study of homeless women in shelters and on the streets, Los Angeles, CA	Homeless, 18–65 years old, having an intimate partner or friend willing to participate	Blood	..	884	..	..
Herndon et al,^25^ 2003	USA	Female	Homeless women in shelters and soup kitchens in Los Angeles, CA	Homeless women in shelters and soup kitchen	Questionnaire	33	970	..	..
Hahn et al,^62^ 2004	USA	Mixed	Homeless people in shelters, free meal programmes, and hostels, San Francisco, CA	Homeless adults	Blood	..	799	..	..
Robertson et al,^65^ 2004	USA	Mixed	Homeless people from shelters, free meal programmes, and hotels, San Francisco, CA	Homeless adults	Blood	42	2508	..	Injection drug use, being male, and white race were positively associated with prevalence; being older than 30 years was negatively associated
Brouqui et al,^60^ 2005	France	Mixed	All homeless adults in two shelters in Marseilles	Homeless adults in shelters	Blood	43	889	..	..
Grimley et al,^61^ 2006	USA	Male	All homeless adults in three shelters in two cities	Homeless adults	Oral fluid	35	285	..	..
Beijer,^50^ 2007	Sweden	Mixed	All homeless people in contact with the Social Services Unit for Homeless People in Stockholm	Homeless adults	Blood	42	2285	..	
Brito et al,^52^ 2007	Brazil	Mixed	Homeless adults who use shelters, São Paulo	Homeless adults aged 18 years or older without psychiatric disturbances who use shelters	Blood	40	330	<1 year (39%), >5 years (13%)	..
Bucher et al,^14^ 2007	USA	Mixed	Homeless people from all homeless shelters and free meal programmes in San Francisco, CA	Homeless adults	Blood	..	681	>1 year (69%)	..
Burström et al,^21^ 2007	Sweden	Mixed	Homeless people in shelters and institutions and rough sleepers in Stockholm	Homeless adults	Questionnaire	48	155	>10 years (40%)	..
Forney et al,^24^ 2007	USA	Mixed	Homeless people in shelters and soup kitchens in San Francisco, CA	Homeless adults	Questionnaire	42	218	..	..
Talukdar et al,^66^ 2007	India	Male	Homeless men living in public space in 30 days in Kolkata	Homeless men aged 18–49 years	Blood	28	493	..	Circumcision was negatively associated with prevalence
O'Carroll et al,^22^ 2008	Ireland	Mixed	All homeless people living in temporary accommodation in Dublin	Homeless adults in hostels and bed and breakfasts	Questionnaire	..	345	..	..
Vahdani et al,^59^ 2009	Iran	Male	Homeless people in institutions of the municipal authorities, shelter homeless, and rough sleepers in Tehran	Homeless, in a shelter	Blood	45	202	Mean 502 days (range 10–3700)	..
Fogg et al,^23^ 2010	USA	Mixed	Homeless people from shelters in the six states in New England	Homeless adults	Questionnaire	..	316	..	..
Laporte et al,^26^ 2010	France	Mixed	Homeless shelters in Paris	Homeless adults	Questionnaire	..	840	Mean 8·5 years in men and 4·3 years in women	..
Wenzel et al,^27^ 2011	USA	Male	Homeless men from meal programmes in Skid Row area of Los Angeles, CA	Homeless heterosexually active men	Questionnaire	..	305	..	Having an HIV-positive partner or several partners was positively associated with prevalence
Colson et al,^53^ 2011	France	Male	Two homeless shelters in Marseilles	Homeless adults	Blood	41	220	..	..

Of the 17 reports for tuberculosis, ten included data for men (n=35 102);^9^, ^20^, ^35^, ^37^, ^38^, ^41^, ^42^, ^46^, ^47^, ^48^ the other seven were mixed-sex samples (8503).^36^, ^39^, ^40^, ^43^, ^44^, ^45^, ^49^ In the surveys with mixed-sex samples, 83% of participants were men (weighted average). Five reports were from the USA (n=32 389),^9^, ^20^, ^38^, ^41^, ^43^ four from the UK (5167),^37^, ^42^, ^44^, ^47^ three from France (1075),^35^, ^40^, ^49^ and one each from Germany^39^ (2308),^39^ Sweden (1704),^36^ Spain (394),^46^ Poland (305),^45^ and Japan (263).^48^ Tuberculosis was diagnosed by chest radiography in 12 studies^9^, ^35^, ^37^, ^39^, ^40^, ^42^, ^44^, ^45^, ^46^, ^47^, ^48^, ^49^ (9977) and by sputum culture in two^41^, ^43^ (2891); the method of diagnosis was unknown in the remaining three (30 737).^20^, ^36^, ^38^

**Figure 1:**
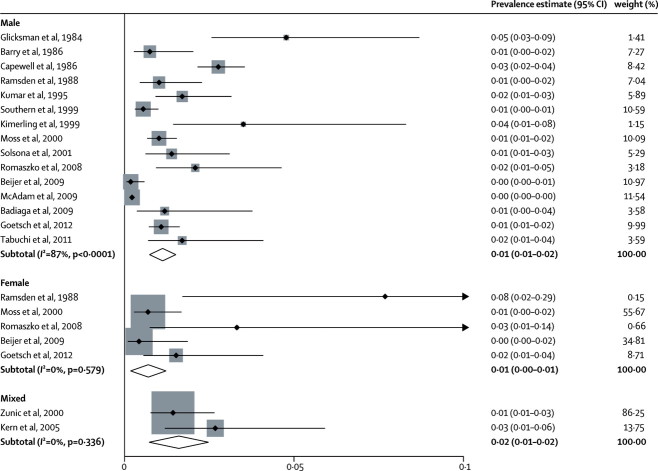
Estimated prevalence of tuberculosis in homeless people Weights are from random-effects analysis. For the mixed and female samples, shading represents, and is proportional to, study weight.

Estimates of tuberculosis prevalence ranged from 0·2% to 7·7% (figure 1); heterogeneity was substantial (χ^2^=126, p<0·0001; *I*^2^=83%, 95% CI 76–89). The random-effects pooled prevalence was 1·1% (95% CI 0·8–1·5). In individual variable metaregression analysis, the prevalence of tuberculosis was higher in studies in which chest radiography was used for diagnosis (p=0·047) than in those in which other diagnostic methods were used; high general population prevalence was related to high prevalence in homeless people (p=0·039; table 4), but the relation did not remain significant after multivariate metaregression.

**Table 4:** Univariate metaregression for prevalences of tuberculosis, hepatitis C virus, and HIV in homeless people

	**Metaregression coefficient (%)**	**95% CI**	**p**
**Tuberculosis**			
Year of publication	−0·04	−0·08 to 0·01	0·065
Sex (male *vs* female)	−0·11	−0·80 to 0·58	0·739
Country (USA *vs* other)	−0·33	−1·22 to 0·56	0·452
Diagnosis (chest radiography *vs* other)	0·74	0·01 to 1·47	0·047
Sample size (>500 *vs* ≤500)	0·76	−0·13 to 1·66	0·091
Sample size, continuous	−0·0004	−0·00009 to 0·001	0·139
Population prevalence (per 100 000)	0·05	0·003 to 0·11	0·039
**Hepatitis C virus**			
Year of publication	−1·20	−3·07 to 0·67	0·191
Sex (male *vs* female)	−1·01	−10·68 to 8·65	0·827
Country (USA *vs* other)	2·29	−9·08 to 14·87	0·615
Diagnosis (blood test *vs* other)	−11·94	−24·49 to 0·61	0·061
Sample size (>500 *vs* ≤500)	1·88	−11·69 to 15·45	0·773
Sample size, continuous	−0·04	−0·11 to 0·09	0·509
Population prevalence (per 100 000)	0·003	−0·006 to 0·01	0·444
**HIV**			
Year of publication	−0·34	−0·66 to −0·02	0·037
Sex (male *vs* female)	−0·80	−3·11 to 1·51	0·488
Country (USA *vs* other)	3·18	−0·19 to 6·16	0·038
Diagnosis (blood test *vs* other)	1·51	−1·88 to 4·89	0·371
Sample size (>500 *vs* ≤500)	1·90	−1·26 to 5·06	0·229
Sample size, continuous	0·00007	−0·03 to 0·04	0·969
Population prevalence (per 100 000)	0·002	−0·01 to 0·01	0·640

**Figure 2:**
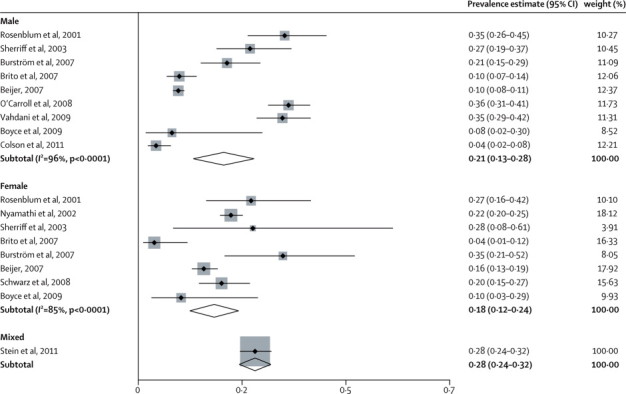
Estimated prevalence of hepatitis C virus infection in homeless people Weights are from random-effects analysis. For the mixed study, shading represents, and is proportional to, study weight.

Three reports in men (n=765),^22^, ^53^, ^59^ two in women (1045),^54^, ^58^ and seven in mixed-sex samples (3581) included data for hepatitis C virus infection.^21^, ^50^, ^51^, ^52^, ^55^, ^56^, ^57^ 77% of participants in mixed-sex samples were men (weighted average). Five reports were from the USA (1758),^51^, ^54^, ^55^, ^57^, ^58^ two from Sweden (2440),^21^, ^50^ and one each from Ireland (343),^22^ Brazil (330),^52^ France (220),^53^ Iran (202),^59^ and the UK (98).^56^ Diagnosis of hepatitis C virus infection was based on blood analyses in nine of the investigations (4795),^50^, ^51^, ^52^, ^53^, ^54^, ^55^, ^57^, ^58^, ^59^ questionnaires in two (498),^21^, ^22^ and PCR analysis of oral fluids in one (98).^56^ Prevalence ranged from 3·9% to 36·2% (figure 2), with substantial heterogeneity between the estimates (χ^2^=354, p<0·0001; *I*^2^=95%, 95% CI 94–96). The random-effects pooled prevalence of hepatitis C virus infection was 20·3% (95% CI 15·5–25·2). None of the factors we explored further was significantly associated with heterogeneity on metaregression (table 4).

Of the 22 reports for HIV infection, five had data for men (n=1505),^27^, ^53^, ^59^, ^61^, ^66^ two for women (1854),^25^, ^54^ and 15 for mixed-sex samples (11 457).^10^, ^14^, ^21^, ^22^, ^23^, ^24^, ^26^, ^50^, ^52^, ^55^, ^60^, ^62^, ^63^, ^64^, ^65^ In the surveys with mixed-sex samples, 69% of individuals were men (weighted average). 13 studies were from the USA (9057),^10^, ^14^, ^23^, ^24^, ^25^, ^27^, ^54^, ^55^, ^61^, ^62^, ^63^, ^64^, ^65^ three from France (1949),^26^, ^53^, ^60^ two from Sweden (2440),^21^, ^50^ and one each from India (493),^66^ Ireland (345),^22^ Brazil (330),^52^ and Iran (202).^59^ HIV was diagnosed on the basis of blood analyses in 14 reports (11 382),^10^, ^14^, ^50^, ^52^, ^53^, ^54^, ^55^, ^59^, ^60^, ^62^, ^63^, ^64^, ^65^ questionnaires in seven (3149),^21^, ^22^, ^23^, ^24^, ^25^, ^26^, ^27^ and PCR analysis of saliva in one (285).^61^

**Figure 3:**
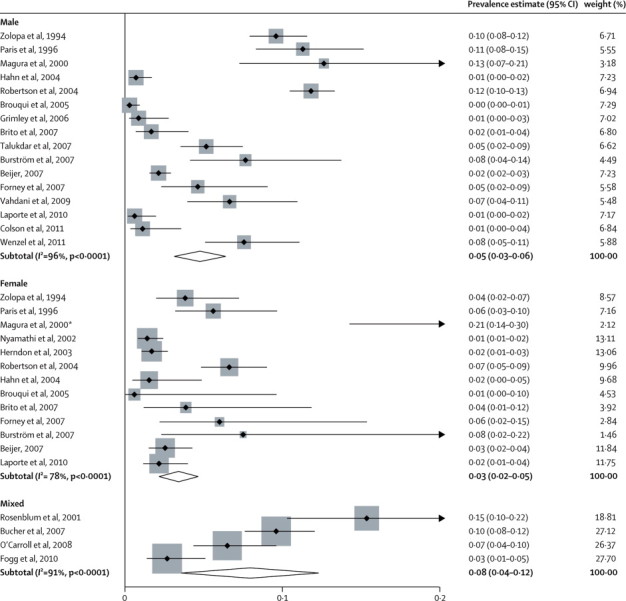
Estimated prevalence of HIV infection in homeless people Weights are from random-effects analysis. For the mixed studies, shading represents, and is proportional to, study weight. *The point for the prevalence estimate is outside the range of the graph.

Estimates of the prevalence of HIV infection ranged from 0·3% to 21·1% (figure 3); heterogeneity was pronounced (χ^2^=541, p<0·0001; *I*^2^=94%, 95% CI 93–95). The random-effects pooled prevalence was 4·7% (95% CI 3·6–5·8). In univariate metaregression analyses, older studies had higher prevalences than did newer studies (p=0·037), and prevalence was higher in studies from the USA (p=0·038) than in those from the rest of the world (appendix); however, these findings did not remain significant after multivariate metaregression.

Prevalences of tuberculosis and hepatitis C and HIV infection in US and European studies seemed similar to overall prevalences, although no statistical comparisons were done. For tuberculosis (excluding a study from Japan^48^), estimates ranged from 0·2% to 7·7%, with substantial heterogeneity (χ^2^=123, p<0·0001; *I*^2^=84%, 95% CI 76–89). The random-effects pooled prevalence was 1·1% (95% CI 0·8–1·4). For hepatitis C virus infection (excluding a study from Brazil^52^ and another from Iran^59^), estimates ranged from 4·3% to 36·2%, with substantial heterogeneity (χ^2^=296, p<0·0001; *I*^2^=95%, 95% CI 94–97). The random-effects pooled prevalence was: 21·4% (95% CI 15·9–26·8). For HIV infection (excluding studies from Brazil,^52^ India,^66^ and Iran^59^), estimates ranged from 0·3% to 21·1%; heterogeneity was substantial (χ^2^=524, p<0·0001; *I*^2^=95%, 95% CI 93–96). The random-effects pooled prevalence was 4·8% (95% CI 3·6–6·0). Use of arcsine-transformed estimates of prevalence made little difference to the overall random-effects estimates, which were themselves shown to be notably different (closer to 50%) from the fixed-effects estimates (in which smaller prevalences have smaller SEs and thus greater weight than they would have in random-effects estimates).

As part of our sensitivity analyses, we excluded one large tuberculosis study;^20^ the prevalences did not change, whereas the random-effects pooled prevalence rose slightly to 1·2% (95% CI 0·8–1·6). For infection with hepatitis C virus, when two studies were excluded in which diagnosis was based on questionnaires^21^, ^22^ and a third in which it was based on analysis of oral fluid,^56^ estimates of prevalence ranged from 3·9% to 35·3% with substantial heterogeneity (χ^2^=257, p<0·0001; *I*^2^=95·3%, 95% CI 93·5–96·7). When we excluded these studies, the random-effects pooled prevalence fell to 17·5% (95% CI 12·4–22·5). For HIV infection, when seven questionnaire studies^21^, ^22^, ^23^, ^24^, ^25^, ^26^, ^27^ and one study based on analysis of oral fluid^61^ were excluded, estimates of prevalence ranged from 0·3% to 21·1% with substantial heterogeneity (χ^2^=494, p<0·0001; *I*^2^=95·7%, 95% CI 94·6–96·7). The random-effects pooled prevalence increased to 5·4% (95% CI 3·9–6·8).

**Figure 4:**
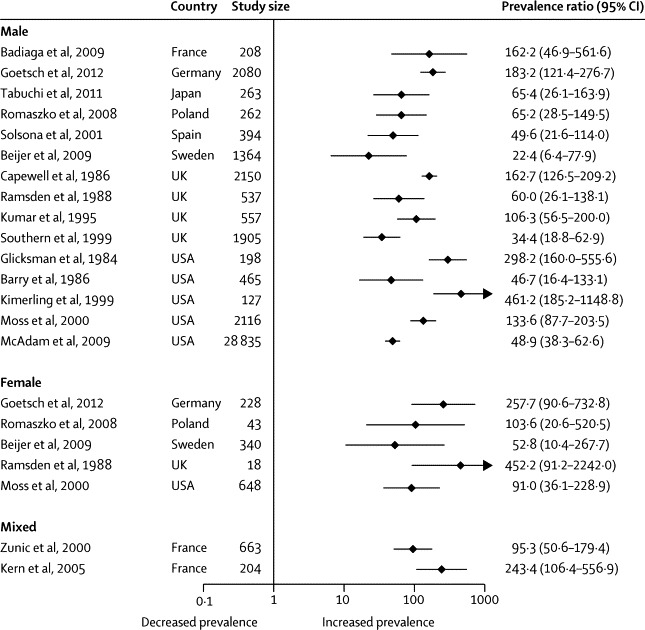
Prevalence ratios of tuberculosis in homeless people versus same-country general populations

**Figure 5:**
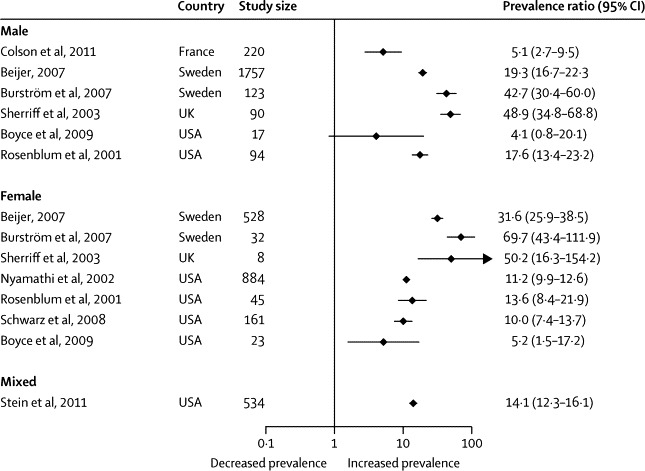
Prevalence ratios of hepatitis C virus infection in homeless people versus same-country general populations

**Figure 6:**
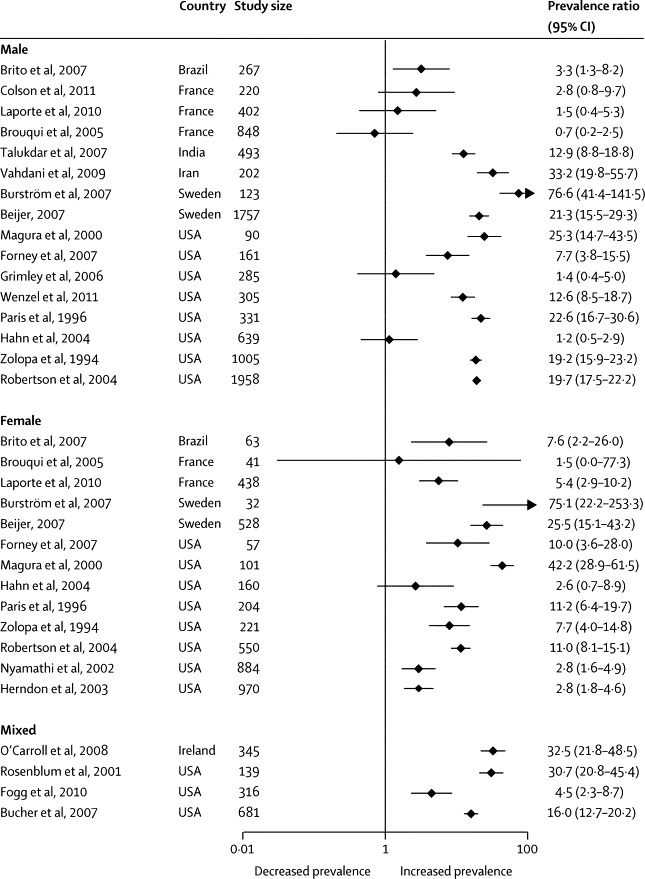
Prevalence ratio of HIV infection in homeless people versus same-country general populations

Prevalence ratios ranged from 34 to 452 for tuberculosis (figure 4), 4 to 70 for hepatitis C virus infection (figure 5), and 1 to 77 for HIV infection (figure 6). Heterogeneity was substantial in all cases (*I*^2^>80%).

## Discussion

Our systematic review and meta-analysis of tuberculosis, hepatitis C virus, and HIV prevalences in homeless people identified 43 studies of 59 736 individuals. Our main finding was that, of these three infectious diseases, hepatitis C virus infection had the highest prevalence in homeless populations and tuberculosis the lowest. Additionally, we noted substantial heterogeneity between studies, suggesting the need for locally based studies to inform service planning and public health measures.

The main implication of our results is that the identification and management of infections should be integral to the planning and development of services for homeless people, which is further emphasised by the risks of contagion to the rest of the community. 2012 clinical guidelines from the UK National Institute for Health and Clinical Excellence^67^ showed that screening and treatment of tuberculosis is cost saving (£20 000 [US$32 000] per quality-adjusted life year) in homeless populations with a tuberculosis prevalence of 0·25% or higher. 15 of 17 studies in our systematic review had higher prevalences than this cutoff, suggesting that universal screening of homeless populations should be considered. True prevalence could be higher still, because subclinical tuberculosis cannot be detected by chest radiography (used in at least 12 of the 17 studies included), the use of which is partly dependent on patients presenting with clinical symptoms.^68^ By contrast, the true prevalence of hepatitis C virus could be lower than that established in our study because positive serology can also be an indicator of past infection. However, both past and active infections are potentially infective^69^ and therefore carry a public health risk.

Our heterogeneity analyses generated several potentially important findings. For tuberculosis, chest radiography was associated with significantly higher prevalence than were other diagnostic methods. This finding might be because of the lower sensitivity of sputum analysis compared with chest radiography.^68^ Prevalence of tuberculosis in homeless people was positively associated with prevalence in the general population, but this relation did not hold for hepatitis C virus and HIV. This result is potentially important from a public health perspective because it suggests that general population measures to reduce rates of hepatitis C virus and HIV infections might not translate into lower prevalences in homeless people. Older studies and those from the USA showed significantly higher prevalences of HIV infection than did newer studies and those from elsewhere. The substantial heterogeneity for all three infections suggests that caution is necessary when pooled estimates are used and emphasises the need for careful description of samples and diagnostic methods in surveys. However, for hepatitis C virus and HIV infections, our sensitivity analyses showed that our overall results were not materially different when we included only studies in which diagnosis was blood based. Characteristics that we did not test might have been associated with heterogeneity, such as length of homelessness or age at onset of homelessness, and future research should describe samples in further detail.

Previous reviews of infectious diseases in homeless people include a 2001 narrative review,^12^ and a 2011 systematic review^30^ of hepatitis C virus infection in the USA that reported a higher prevalence than that in our review because it included selected populations of homeless people—eg, those with co-occurring HIV infection or other medical illnesses and those who misuse substances.

We did not limit studies by country in our inclusion criteria. All but four studies were done in Europe or the USA, showing the need for further research in low-income and middle-income countries (some of which will be undergoing rapid urbanisation). Of the 43 studies included in our systematic review, only eight include prevalence estimates for more than one of the diseases that we investigated. An important limitation of our work is that we did not include other infections because our initial scoping search did not identify many relevant studies and we wanted to focus on the infections that arguably have the largest effect on public health. High rates of infection with hepatitis A and B viruses, diphtheria, and influenza have been reported in homeless people.^12^ Furthermore, a narrative review by Raoult and colleagues^12^ has shown the morbidity associated with foot problems and skin infections—eg, scabies, body lice, and louse-transmitted infections. Further research should assess other infectious diseases, especially if these additional investigations have little cost.

Another limitation is that our systematic review is based on cross-sectional designs, and therefore inferences about causality cannot be made. However, several included studies reported risk factor information, and the role of injection drug use should be examined further. We did not identify any longitudinal studies, which would be practically difficult but important for future research because they would provide information on the development of infectious diseases and especially risk factors and mediators.

Prevalence ratios suggest that, in the USA, tuberculosis is at least about 46 times more common in homeless than in general populations; the prevalence of hepatitis C virus infection is increased about four times. Prevalence ratios were also increased for the HIV studies, but not to the same extent. These ratios contrast with the prevalence data, in which hepatitis C virus infection typically has the highest absolute rates of infection. A more direct comparison than these prevalence ratios would be studies that use the same sampling methods, diagnostic approaches, and interviewers that are used to estimate prevalences in the homeless sample to establish prevalences of infection in a general population sample; we only identified one such study.^36^

Prevalences in homeless people could be compared with those in other high-risk groups within the same geographical regions—eg, prisoners. Incarcerated people have increased rates of morbidity and mortality, especially for infectious diseases,^70^ and targeted interventions could have substantial public health effects.^71^ Mean rates of tuberculosis were higher in homeless people than in prisoners, in whom notification rates for tuberculosis are reported to vary from 0 to 1167 per 100 000.^72^ Similarly high rates have been reported for hepatitis C virus infection, with studies of seropositivity from 14 countries showing antibodies to hepatitis C virus in 2–58% of prisoners and typical rates of 30–40%.^73^ Estimates of the prevalence of HIV infection in prisoners in high-income countries range from 0% to 7·5%, and in the USA, prevalence was estimated at 1·5% in 2007–08.^70^, ^74^ Few comparative data exist for prisoners in low-income and middle-income countries.^75^

Many people transition between prison and being homeless,^76^ suggesting sizeable overlap between these estimates. 2012 National Institute for Health and Clinical Excellence guidelines^67^ recommend that these marginalised groups should be screened simultaneously for tuberculosis, hepatitis C virus, and HIV, and, when necessary, patients should be helped to overcome barriers to completing screening and treatment, such as transport, housing, nutrition, and immigration status.^67^

Screening for tuberculosis should be done through active case-finding—ie, should not be restricted to symptomatic people presenting to health services, which happens less and later in marginalised groups than in general populations.^67^ Other measures, including syringe and needle exchange programmes, free condom distribution, and treatment of related infections (particularly scabies,^77^ body lice,^78^ and louse-borne infections^60^) have been recommended.^79^ Yearly snapshot interventions,^60^ inpatient treatment of specific infections because of the risk of non-adherence, and first-aid centres in large cities should be considered.^12^ Whenever possible, screening should follow best-practice guidelines; diagnosis should be based on chest radiography and analysis of oral fluid for tuberculosis^80^ and blood-based testing for hepatitis C virus^69^ and HIV.^81^ In addition to these targeted measures, reduction of the inequalities faced by homeless people in overall social determinants of health could be part of a wider public health strategy to address infections in some countries.^82^ Other population-based approaches might include housing policies^83^ and equal access to health care.^84^
