# Supplementary material for: Prevalence of tuberculosis, hepatitis C virus, and HIV in homeless people: a systematic review and meta-analysis
Source: Lancet Infect Dis. 2012 Nov;12(11):859–70. doi: 10.1016/S1473-3099(12)70177-9 (PMC3494003; doi:10.1016/S1473-3099(12)70177-9)
Supplement: Supplementary appendix [file mmc1.pdf]

THE LANCET Infectious Diseases

Supplementary webappendix

This webappendix formed part of the original submission and has been peer reviewed. We post it as supplied by the authors.

Supplement to: Beijer U, Wolf A, Fazel S. Prevalence of tuberculosis, hepatitis C virus, and HIV in homeless people: a systematic review and meta-analysis. *Lancet Infect Dis* 2012; **12:** 859–70.

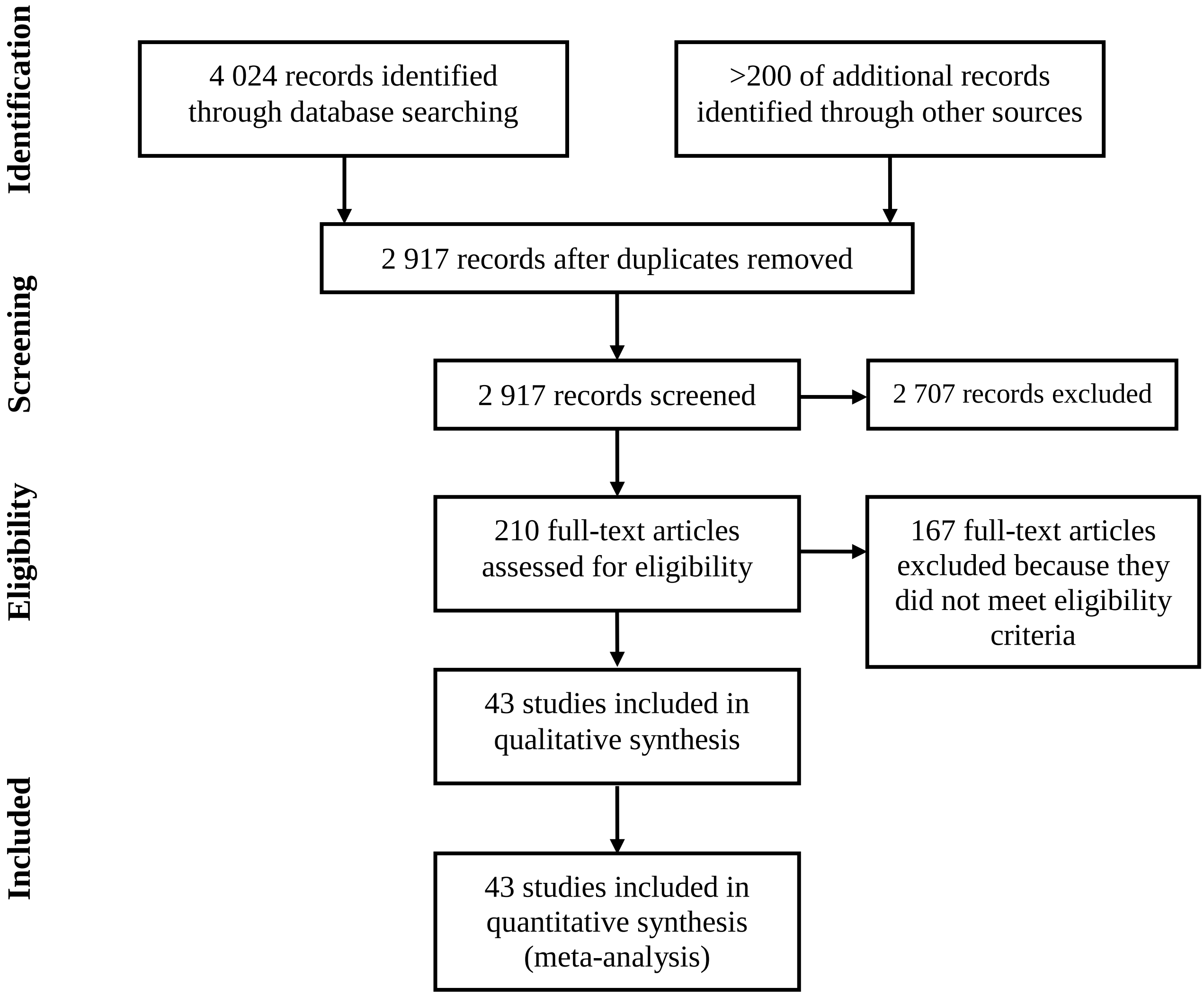

Flow-chart documenting the systematic search conducted to identify included studies

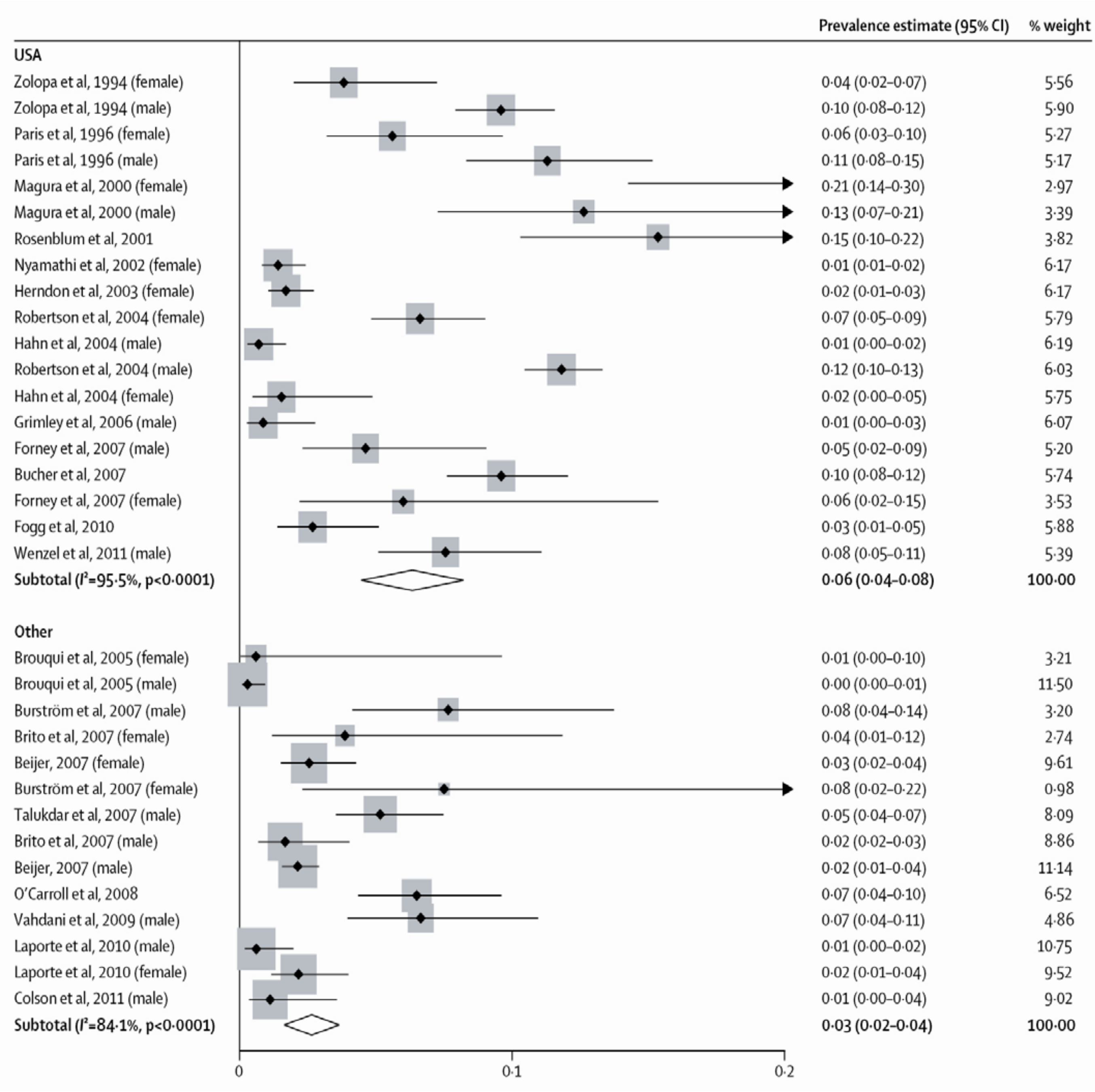

Figure: **Prevalence of HIV infection in homeless people in the USA compared with that in other countries**

Weights are from random effects analysis.
